# Mothers' involvement in a school-based fruit and vegetable promotion intervention is associated with increased fruit and vegetable intakes – The Pro Children study

**DOI:** 10.1186/1479-5868-5-48

**Published:** 2008-10-15

**Authors:** Saskia J Te Velde, Marianne Wind, Carmen Perez-Rodrigo, Knut-Inge Klepp, Johannes Brug

**Affiliations:** 1 EMGO institute, VU University Medical Center, Van der Boechorststraat 7, 1081 BT, Amsterdam, The Netherlands; 2 Department of Nutrition, Faculty of Medicine, University of Oslo, PO Box 1046, Blindern, 0316 Oslo, Norway; 3 Community Nutrition Unit, Department of Public Health, Luis Briñas 18; 4 Planta E 48013 Bilbao, Spain; 4 Department of Nutrition, Faculty of Medicine, University of Oslo, PO Box 1046, Blindern, 0316 Oslo, Norway; 5 Brug J, EMGO institute, VU University Medical Center, Van der Boechorststraat 7, 1081 BT, Amsterdam, The Netherlands; 6 Department of Public Health, Erasmus University Medical Center, Rotterdam, The Netherlands

## Abstract

**Background:**

Several school-based fruit and vegetable interventions include activities to involve parents, but not much is know about the effectiveness of such a family component on child and parent intake levels. The current study evaluated the effects of the multi-component school-based intervention, 'the Pro Children Study', on mothers' intake levels. Furthermore, associations between level of involvement in the project and improvement in the mothers' intake levels were assessed.

**Methods:**

Effect was evaluated in a cluster randomized controlled trial in Spain, Norway and the Netherlands among mothers of 11-year-olds. Of the 1253 mothers with complete data at baseline, 754 and 476 had complete data at first and second follow-up respectively. Fruit and vegetable intake, level of involvement and demographic variables were assessed by a parental questionnaire. Data was analyzed using multilevel regression analyses.

**Results:**

Results showed no effect of the intervention on mothers' fruit and vegetable intake after one year and two year follow-up. Participation rate for the different activities varied by activity and by country, e.g. 3.7–9.4% visited the website, while 26.4–72.6% of the mothers participated in the home work assignments. Results further showed that higher involvement levels were associated with higher intake at follow-up.

**Conclusion:**

The Pro Children Intervention could not increase the fruit and vegetable consumption of the mothers of participating pupils, which might be explained by the low involvement in the project. More research is needed to increase mothers' involvement in school-based interventions.

## Background

A large proportion of the Western populations does not meet the recommendations for fruit and vegetable intake [1-3], while epidemiological studies show beneficial effects of diets high in fruit and vegetables [4-6]. Therefore, several programs have been developed to encourage fruit and vegetable intake. The Pro Children Intervention is one example, which was implemented and evaluated in a cluster randomized controlled trial in three European countries, namely Norway, Spain and the Netherlands [7], and which has proven to be successful in increasing fruit and vegetable intake among 11-year-old schoolchildren [8].

The Pro Children Intervention was a multi component intervention consisting of a class room component, a school component and a family component in which parents were encouraged to participate in various activities [9]. Since parents were encouraged to be involved in the project and children were taught skills to ask for fruit and vegetables at home, the intervention may also have improved fruit and vegetable intakes of the parents.

Several school-based fruit and vegetable interventions comprising family components have been described in the literature [10], however, only one described effects in intake among parents [11]. The High 5 Intervention resulted in higher fruit and vegetable consumption in parents in the experimental group compared to the control group at first follow-up at 12 months, but this effect was not sustained at second follow-up, one year later. A Social marketing inspired intervention including newsletters and parent events (duration 4 weeks) resulted in more frequent serving of fruit and vegetables during dinner and more frequent serving of fruit as a snack among the parents at post-test, compared to the pre-test [12]. If school-based interventions are successful in promoting fruit and vegetable intake among parents, this will be an additional positive effect, which might be relevant for policymakers deciding upon large scale implementation of school based interventions. Moreover, parents who increase their own fruit and vegetable intake may also serve as better role models for their (other) children [13-15]. Such an effect among parents is most likely if parents are actively involved in the school-based project.

In the Pro Children intervention study, data is available on fruit and vegetable intake of the parents of the participating pupils, therefore, the aim of the current study was to evaluate the effects of a school based intervention on parents' fruit and vegetable intake after one year and two year follow-up. A second aim was to assess whether a potential effect was mediated by the level of involvement in the project.

## Methods

### The intervention

The Pro Children intervention consisted of different components: a classroom component, a school component, a family component and one optional component, which differed slightly by intervention site, as described in more detail elsewhere [8,9].

The *classroom curriculum *consisted of school education materials containing of a set of 16 worksheets with guided activities and a web-based computer-tailored feedback tool. These educational classroom activities addressed the knowledge of recommended intake levels, awareness of the children's own intake, taste and preferences for different kinds of fruits and vegetables and specific skills to prepare or ask for fruit/vegetables. The *school component *consisted of the provision of fruit and vegetables, either for free (Netherlands), by means of a subscription program (Norway) or as part of the school meals (Spain). Providing fruit and vegetables increased the school availability, accessibility and exposure to fruit and vegetables. The *family component *encouraged parents to be involved in the project by means of their children's homework assignments, parental newsletters and a parent version of the web-based computer-tailored tool that enabled them to get personalized feedback on their own fruit and vegetable intake levels [16]. The newsletters included a variety of information on fruit and vegetables, announcements about ongoing activities and tips for parents to encourage their children to eat more fruit and vegetables. These activities tried to influence the parents as important role models, by advising them on how to support their children in eating fruit and vegetables, and how to increase home availability and accessibility. The computer-tailored tool addressed determinants of fruit and vegetable intake in order to increase fruit and vegetable consumption of the parent [16]. As all other components, this component was most intense during the first year. During the second year only a free fruit and vegetable cookery book, continuation of a computer-tailored fruit and vegetable intervention and two news letters were offered.

The *optional component *aimed to encourage community participation in the Pro Children Project. In the Netherlands and Norway the local media was used to raise awareness. In Spain, school health services participated in the project and counseled students during their regular health visits.

### Design

The effects of the intervention were examined in a group-randomized trial design among fifth and sixth graders and their parents, from 62 schools in three European counties, i.e. in the Buskerud County of Norway, in Rotterdam, the Netherlands, and in the Bilbao region, Spain. In each country the schools were randomly assigned to an intervention or control group. Surveys among all participating children and one of their parents were conducted prior to the intervention (September 2003), immediately after (May 2004) and at the end of the following school year (May 2005) [7,8].

Sample size calculations were performed to identify the number of children needed to show a significant intervention effect of 20% [8].

### Measures

Parental fruit and vegetable consumption, parental involvement in the intervention and demographic variables were measured by parental questionnaires. These questionnaires were given to the participating children to take home. Parents themselves could decide which parent was to complete the survey. This led to a gender-related selection bias as the questionnaire was primarily filled out by the parent traditionally responsible for the child's care, i.e., the mothers.

Primary outcome measures were the total intake of fruit and vegetables and the intake of fruit and vegetables separately, i.e. the amount (grams) of fruit and/or vegetables consumed on the day prior to the day of data collection (24-hour recall). The 24-hour recall instrument has previously been described and validated by Kristjansdottir [17]. Briefly, the pre-coded 24-hour recall section informed about yesterdays fruit and vegetable consumption by questions about fruit and vegetable intake referring to three different time intervals: morning/midmorning, lunch time/afternoon and dinner/later in the evening. In each of the three periods of the day, participating parents were asked, using an open question, what they had eaten during that period the day before. This was done to prompt the participant's memory to that specific period of the day. Then there were specific precoded questions on juice, fruits and vegetables. Amounts were indicated in terms of the number of pieces, slices or portions eaten, and standards were defined for these units. Total fruit and/or vegetable intake were calculated by summarizing all answers. Potatoes and dried fruit were not included.

As part of the process evaluation of the Pro Children Intervention, parental involvement was assessed by asking the parents ('yes' = 1, 'no' = 0), whether they had visited the Pro Children website, whether they had conducted the computer-tailored tool for adults, whether they had seen at least two of the three newsletters, whether they had helped their children doing three homework assignments that specifically asked for their input (going to the supermarket with the child, prepare a vegetable dish together with the child and help the child to make a recipe about fruit and vegetables), and whether they went to the parents meeting at school. Of these seven activities a composite score was calculated (range between 0–7). Educational level of the parent, having a paid job, immigrant status, living alone or with a partner, and the gender of the child were considered as potential confounders in the effect analyses. Educational level was assessed in the parental questionnaire, and was categorized based on the number of years of education completed by the parent: less than seven years, between seven and nine years, between ten and twelve years and more than twelve years. Immigrant status was assessed by questions on the country of birth. When the parent was not born in the country under study, (s)he was considered as an immigrant.

### Respondents and preliminary data handling

**Figure 1:**
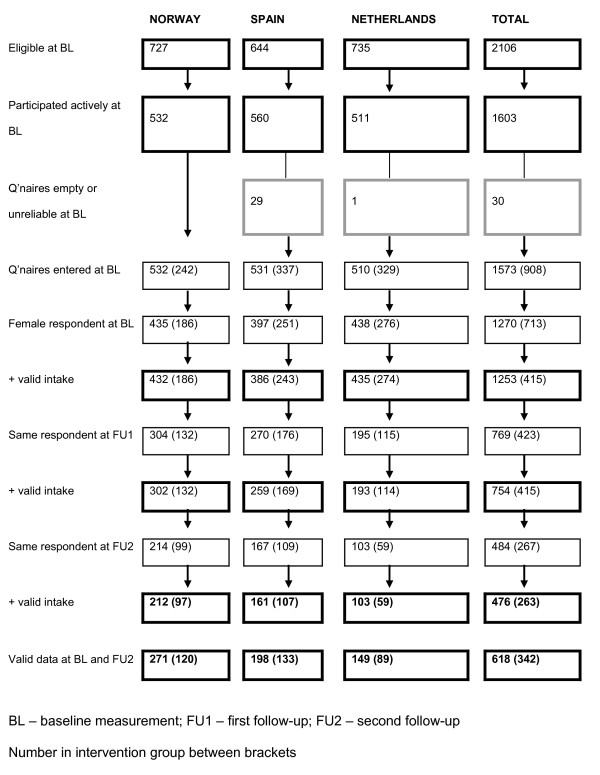
Flowchart of the inclusion procedure for the total sample and by country (numbers between brackets are the numbers in the intervention group).

At the start of the study a total of 2106 children and parents were eligible for participation. At baseline 1843 children were present on the day of the data collection and could bring a parent questionnaire home, and 1603 parents returned a questionnaire of which 30 were empty (26) or unreliable (4), so finally 1573 (74.7% of eligible sample) questionnaires were entered at baseline. At first follow-up 1276 questionnaires were entered and at second follow-up 1123 questionnaires. As mentioned before, parents could decide for themselves who completed the questionnaires. As a consequence, the three questionnaires were not always completed by the same person. In the vast majority of the cases, all three questionnaires were completed by the mother or female caregiver (72.8% in Spain – 87.8% in the Netherlands). In only 5.4% of the cases all three questionnaires were completed by the father or male caregiver. Therefore, we only included female respondents, i.e. mothers, stepmothers or female guardians, in the analyses. Figure 1 shows in more detail the inclusion of the participants in the current study. In the Netherlands, a large proportion (51%) of the parents did not answer the question on what his/her relation to the child was at first and second follow-up, so we could not identify the gender of the parent and cases were therefore excluded from the analyses. Thus, at the first follow-up 762 parents with complete data on gender and their fruit and vegetable intake could be included (35.8% of eligible sample), while 618 mothers had complete data at baseline and the second follow-up (29.3% of eligible sample). Only 483 parents had complete data on all three time points (22.9% of eligible sample).

Of the mothers with complete data at baseline and the first follow-up who belonged to the intervention group (n = 415), 385 completed at least six of the seven questions on parental involvement in the Pro Children activities.

As some respondents had missing values on some of the other variables, the numbers presented in the tables may vary slightly.

### Statistical data analyses

Descriptive statistics were used to describe key participant characteristics and variable scores for the intervention and control group at baseline and follow-up measurements.

To assess potential dropout bias both at first and second follow-up, multiple logistic regression analysis was conducted with dropout as dependent variable and country, intake at baseline, treatment condition, and demographic variables as independent variables.

As a consequence of the study design pupils and their parents were nested within schools and a high probability of interdependence can be expected between the parents belonging to the same school. To take this into account, multilevel analyses with random intercepts were conducted in MlwiN 2.02 [18] to assess the effects of the intervention on fruit and vegetable intake, while controlling for gender, country and baseline value of the outcome measure.

Effects of the intervention on parental fruit and vegetable intake was separately assessed for first follow-up and for second follow-up using multi level regression analyses with group (control = 0, intervention = 1) as the independent and fruit and vegetable intake as the dependent variable. Regression analyses was appropriate to use, since the distribution of the residuals was normal.

Main analyses were conducted on samples with either complete data at baseline and first follow-up or with complete data at baseline and second follow-up. Potential confounding by the educational level of the parent, having a paid job, immigrant status, living alone or with a partner, and the gender of the child, was assessed. When these co-variates did not confound the findings, results were presented from the regression models without these variables.

Intention-to-treat analyses (last value carried forward method) was conducted as a sensitivity analyses for the estimated intervention effect.

Effect modification by gender of the child, living alone or with a partner, having a paid job, mothers educational level and immigrant status of the parent were additionally assessed by including an interaction term between the group variable (intervention or control) and the potential effect modifier. In case of significant interaction (p < 0.1), stratified analyses are presented.

In order to assess whether parental involvement in the project mediated the effect of the intervention, no traditional method, such as suggested by Baron & Kenny [19] could be used, since data on parental involvement was only available in the intervention group. Therefore we assessed whether level of involvement in the project was associated with (potential) achieved changes in fruit and vegetable intake between the baseline measurement and the follow-up measurements. To do so, a composite measure of all seven involvement activities was calculated (range 0–7). This measure was used as a categorical variable in the analyses (reference group = low involvement (score < 2), medium involvement (score = 2 or 3), high involvement (score ≥ 4).

## Results

### Drop out analyses

Drop out analyses using a regression model including only country and baseline fruit and vegetable intake as predictors, showed that Dutch mothers were more likely to drop out (OR = 2.80, 2.10–3.72) than mothers from Norway and Spain. Higher fruit and vegetable intake at baseline was also associated with increased odds to drop out between baseline and first follow-up (OR = 1.08, 1.03–1.14 for each 100 gram higher baseline intake).

A regression model additionally including all demographic variables showed that mothers of boys were more likely to drop out (OR = 1.64, 1.17–2.29), and that immigrant mothers dropped out more often (OR = 2.34, 1.62–3.37) than mothers of girls and mothers born in the country of residence. Especially in the Netherlands immigrants in the intervention group dropped out more often than mothers born in the Netherlands (82.1% versus 40.5%, p < 0.001). None of the other demographic variables were associated with drop out rates.

### Descriptive statistics

Table 1 shows descriptive statistics of the demographic variables and baseline intake of fruit and vegetables for the mothers included in the study. As can be seen, a considerable proportion of the Dutch sample consisted of mothers not born in the Netherlands. Overall, the majority of the mothers lived with their partners and with their children, and they reported having paid jobs. Baseline fruit and vegetable consumption did not differ between mothers in the intervention and the control groups.

**Table 1:** Descriptive statistics for demographic variables and fruit and vegetable intake at baseline, first and second follow-up for the control and intervention group separately

	Norway		Spain		Netherlands	
	Intervention group	Control group	Intervention group	Control group	Intervention group	Control group

	← N (%) →					

Immigrant status (born outside research country)	9 (6.8%)	15 (8.8%)	7 (4.4%)	7 (8.0%)	21 (18.4%)	20 (25.3%)

Lives with her partner	108 (81.8%)	141 (82.0%)	149 (87.1%)	80 (87.9%)	95 (83.3%)	65 (82.3%)

Lives with the child participating in the study						

No	0	0	1 (0.6%)	0	0	0
Yes all the time	129 (97.7%)	167 (97.7%)	167 (97.1%)	89 (98.9%)	111 (96.5%)	75 (96.2%)
Yes part of the time	3 (2.3%)	4 (2.3%)	4 (2.3%)	1 (1.1%)	4 (3.5%)	3 (3.8%)

Educational level						

< 7 years	1 (0.8%)	3 (1.8%)	20 (12.0%)	9 (10.6%)	7 (6.1%)	5 (6.6%)
7–9 years	19 (14.4%)	26 (15.3%)	61 (36.5%)	40 (47.1%)	15 (13.2%)	1 (1.3%)
10–12 years	63 (47.7%)	71 (41.8%)	38 (22.8%)	15 (17.6%)	28 (24.6%)	14 (18.4%)
> 12 years	49 (37.1%)	70 (41.2%)	48 (28.7%)	21 (17.6%)	64 (56.1%)	56 (73.7%)

Has a paid job	108 (83.1%)	144 (87.3%)	88 (52.1%)	53 (60.9%)	75 (69.4%)	48 (64.0%)

Gender of the child (boys)	67 (39.6%)	54 (41.9%)	43 (47.8%)	72 (42.6%)	39 (49.4)	42 (36.8)

Intake at baseline	← mean (SD) →					

Total Fruit and vegetable intake (gram/day)	289 (197)	306 (200)	234 (179)	238 (162)	301 (197)	320 (202)

Total Fruit intake (gram/day)	181 (150)	177 (149)	166 (151)	172 (139)	170 (130)	191 (129)

Total Vegetable intake (gram/day)	107 (106)	128 (125)	69 (62)	66 (66)	131 (111)	129 (119)

Only mothers that had valid data at baseline and at first follow-up are included in this table

### Intervention effects on mother's intake

As can be seen in Table 2, no significant intervention effects were observed regarding fruit and vegetable intake of the mothers. Results were not confounded by demographic variables (data not shown). No effect modification by country or any of the demographic variables was observed. The intention-to-treat analyses did not result in other findings (data not shown).

**Table 2:** Unadjusted mean intake values at baseline, first and second follow-up and the results of the multilevel regression analyses showing the effect of the intervention at first and second follow up

	First follow-up (n = 754)				Second follow-up (n = 618)		
	Intake at baseline (mean +/- SD)	Intake at first follow-up (mean +/- SD)	Regression coefficient^1^	95% CI	Intake at second follow-up (mean +/- SD)	Regression coefficient^1^	95% CI

Total fruit and vegetable intake (gram/day)							

Control group	292.3 +/- 193	277.0 +/- 196	9.0	-19.0 – 37.1	283.2 +/- 198	11.9	-20.2 – 43.9
Intervention group	270.8 +/- 193	277.6 +/- 184			287.9 +/- 241		

Total fruit intake (gram/day)							

Control group	180.0 +/- 142	168.4 +/- 135	10.8	-14.8 – 36.4	177.5 +/- 145	9.9	-14.3 – 34.2
Intervention group	172.5 +/- 146	176.5 +/- 132			191.4+/- 185.7		

Total vegetable intake (gram/day)							

Control group	112.2 +/- 114	108.6 +/- 109	-2.7	-16.4 – 11.0	105.7 +/- 104	-0.8	-16.7 – 15.2
Intervention group	98.3 +/- 96	101.1 +/- 97			96.4 +/- 106		

^1 ^Analyses are adjusted for baseline intake, country and clustered design.

Regression coefficients reflects the difference between intervention and control groups

### Parental involvement

Table 3 shows the activities that parents could involve in and how often this was actually done by the mothers included in the current study. Only few mothers visited the Pro Children website (3.7–9.4%) or did the online tailored fruit and vegetable test (0–7.7%). Larger proportions of mothers participated in the home work assignments (26.4–72.6%) or went to the parental meeting at school (6.0–62.6%). However, involvement in these activities varied by country, with highest involvement in Norway and lowest in the Netherlands (see Table 3). This is also expressed in the sum score calculated over all activities. Norway had the highest score (3.4 points, p ≤ 0.001) and the Netherlands the lowest (2.3 points, p < 0.001 with Norway, p = 0.018 with Spain).

**Table 3:** Number of mothers participating in the different involvement activities

	Norway		Spain		Netherlands	
	Responses (N)	Yes (%)	Responses (N)	Yes (%)	Responses (N)	Yes (%)

Mothers in intervention group	132		169		114	
Visit website	127	9.4	164	3.7	109	5.7
Did FV test	105	3.8	95	0	104	7.7
Seen at least two newsletters	126	80.2	138	77.5	106	64.1
Assignment: go to supermarket with child	130	73.1	157	72.6	107	49.5
Assignment: prepare vegetable dish at home with child	130	70.8	153	60.8	108	53.7
Assignment: make a recipe about FV with child	130	47.7	150	58.7	109	26.4
Go to parental meeting at school	131	62.6	151	6.0	105	13.3

	Mean	SD	Mean	SD	Mean	SD

Sum score (range 0–7)	3.4^a^	1.7	2.8^b^	1.4	2.3^c^	1.7

a, b, c – indicate significant differences between the countries (ANOVA, bonforoni post hoc)

Relating this sum score to the mother's intake at first follow-up adjusted for baseline intake, showed positive effects of parental involvement on the mothers' intake at first follow-up (see table 4), but not at second follow-up (data not shown). Results presented in Table 4 show that mothers who participated in 3 to 4 activities (medium involvement) or who participated in more than 4 activities (high involvement) had higher consumption levels than mothers who participated in less than three activities (low involvement). Furthermore, this table also shows that mothers who were more involved had higher intake levels at baseline.

**Table 4:** Unadjusted values for fruit and vegetable intake and adjusted regression coefficients reflecting difference in intake at follow up between the different groups for the level of involvement

	Intake at baseline (mean +/- SD)	Intake at first follow-up (mean +/- SD)	Regression coefficient^1^	95% CI
Total fruit and vegetable intake (gram/day)				

Low involved (< 3)	261.8 +/- 172	247.8 +/- 155	ref	
Medium involved (3–4)	259.7 +/- 197	284.5 +/- 190	43.8	7.3 – 80.4
High involved (≥ 5)	313.0 +/- 206	339.4 +/- 225	94.7	43.6 – 145.9

Total fruit intake (gram/day)				

Low involved (< 3)	173.0 +/- 130	166.6 +/- 122	ref	
Medium involved (3–4)	160.9 +/- 143	175.9 +/- 136	17.1	-9.4 – 43.7
High involved (≥ 5)	191.7 +/- 160	209.6 +/- 142	53.7	16.2 – 91.1

Vegetable intake (gram/day)				

Low involved (< 3)	88.8 +/- 88	81.2 +/- 73	ref	
Medium involved (3–4)	98.8 +/- 94	108.5 +/- 109	27.2	7.0 – 47.4
High involved (≥5)	121.3 +/- 120	129.8 +/- 113	46.0	17.0 – 74.9

^1 ^Analyses are adjusted for baseline intake level, country and clustered design

Regression coefficient reflects difference in intake at first follow-up compared to the reference group adjusted for baseline values.

## Discussion

The current study assessed whether a successful school based multi-component intervention aimed at children, could also positively effect the fruit and vegetable intake of the mothers of the participating pupils. The findings indicate that the intervention did not improve the intake levels of the mothers. This is in contrast with earlier findings in the High Five intervention study reported by Reynolds et al [11], who reported positive intervention effects on parental fruit and vegetable intake in the High Five intervention. The High Five intervention had a family component that consisted of seven homework assignments, brochures, skills building materials (e.g. recipes) and seven interactive lessons to be completed together with the child. Children were also rewarded when these interactive lessons were completed and brought back to the class [11]. Most of these activities are comparable with the activities in the Pro Children intervention, but the Pro Children intervention included fewer activities. Unfortunately, Reynolds et al [11] do not report to what extend parents were really involved in all activities. The current study showed that in general, mothers were not very involved in the Pro Children intervention, although this differed by country, with highest involvement by the Norwegian mothers. Furthermore, the current study showed that the level of involvement was positively associated with changes in fruit and vegetable consumption (Table 4), or at least indicated that the mothers that were involved in the project remained their baseline intake levels. That only a small proportion of the mothers were sufficiently involved in the project, might explain the lack of an overall positive intervention effect on mothers' fruit and vegetable intake. Results suggest a dose response relationship, with more change in those with higher levels of involvement. Another approach to parental involvement was taken by Golan et al [20], who used parents as the sole 'agents of change' in the treatment of childhood overweight and parents were thus actively involved in the project. Their approach was successful in not only reducing childhood overweight but also to improve weight status of the parents and improve eating and physical activity behavior of the parents, showing that an intervention aimed at children's health behavior can also effect parents' behavior.

In addition, the Pro Children consortium [21] showed that parental involvement was also associated with changes in fruit and vegetable intake in the participating pupils. Both findings and conclusions of a review study by Knai et al [10] stress the importance of parental involvement in school based studies promoting fruit and vegetable intake. Parents that are involved in the project are more likely to improve their own intake levels or maintain their relatively high intake levels. Subsequently they will act as better role models [13] and will probably improve the availability of fruit and vegetables at home, which increases the opportunities for children to eat fruit and vegetables. Regretfully, the Pro Children intervention did not succeed in involving the parents in most of the organized activities and assignments. Norwegian schools were most successful in involving the parents, the Norwegian schools were also better in implementing the school curriculum as reported previously [21]. We hypothesized that better implementation was due to a special teacher that could devote his/her time to the implementation of the Pro Children intervention, while in Spain and the Netherlands the teacher responsible for the participating class had to do this in addition to his/her normal tasks. However, the Norwegian schools did not devote more time or effort to get parents involved compared to their Dutch or Spanish colleagues. We speculate that Norwegian parents were more involved as a result of the better implementation of the school curriculum, which might have resulted in Norwegian children talking about the project more often than Dutch or Spanish children. On the other hand, it might also be a cultural difference. Despite the higher involvement of the Norwegian mothers, intervention effects did not differ by country (no significant effect modification by country).

Activities that were most often done by the parents were reading the newsletters and the home assignments, especially the one asking them to prepare a vegetable dish or go to the supermarket together with their child. It might be that these activities were perceived as the easiest ones, or the ones that did not require a big time investment. Completing the tailored fruit and vegetable test at the Pro Children website was only done by a small proportion of the mothers, indicating that this was not a very successful tool to reach the parents. Parents might have thought that completing such a test would take a lot of their time. Internet access might have been an additional issue for some parents, however most households in all three countries have internet access. Future studies should focus on what kind of activities parents prefer in order to increase their involvement. Better insight in parental preferences could make future interventions including parental activities more efficient, since no effort should be put into activities that are not very popular.

To our knowledge, this is the first study investigating not only effects of a school based intervention on parental fruit and vegetable intake, but also assessing the level of actual involvement of the mothers in the organized activities. Furthermore, we aimed to assess whether a potential intervention effect could be mediated by parental involvement. This latter is very important information, since it gives insight in why the intervention was not effective in improving mothers' fruit and vegetable intake and not much is known yet about the effectiveness of parental involvement in school-based studies [22]. Our findings suggest that parental involvement might be a mediator, however, no formal mediation analyses could be conducted. Future research should assess why mothers are involved or not involved, in other words, the next step is to explore determinants of parental involvement in multi-component school-based interventions. With this information we could improve the current intervention.

A limitation of the current study is the high drop-out, especially in the Netherlands. This was mainly caused by the fact that the first question in the questionnaire, asking for the parents relationship with the child, was put on the left page where only one other question was placed. Furthermore, more mothers not born in the Netherlands did not participate in the follow-up measurements, probably due to language issues. Moreover we found that mothers with high intake levels at baseline were more likely to drop out. However, in a regression model also including immigrant status, this was no longer significant. It appeared that immigrants had higher fruit and vegetable intake levels than the native mothers. This high attrition rate may have serious consequences for generalizability of the findings and the external validity of the results. The small number of mothers that was eventually included in the analyses, may also be a reason for not finding significant effects. On the other hand, results were also not close to statistical significance and an increased number would probably not have resulted in positive intervention effects. Furthermore, the current study could not test whether high fruit and vegetable consumers were more likely to be involved in the project or the other way around. Despite these limitations, the findings concerning the association between mother's involvement in the project and changes in fruit and vegetable consumption, give us an indication that parental involvement in school based intervention may be very relevant, not only to improve nutrition behavior of their children, but also to improve their own diets.

In conclusion, the Pro Children Intervention could not increase the fruit and vegetable consumption of the mothers of participating pupils and the involvement of the mothers in the various activities was rather low, which might be an explanation for the lack of effect. Especially because higher involvement in the activities was associated with improvement in fruit and vegetable consumption between baseline and first follow-up. Future research should replicate our findings and explore determinants of parental involvement in school-based interventions and develop strategies to increase parental involvement in order to make interventions (more) successful.

## Competing interests

The authors declare that they have no competing interests.

## Authors' contributions

SJtV performed the analyses, wrote the manuscript and incorporated input from all other authors on the manuscript. MW and CPR coordinated the data collection and implementation of the study and contributed in writing the manuscript. KIK developed and directed the overall study and provide critical comments on the manuscript. JB developed and directed the overall study, and provided critical comments on the manuscript. All authors have read and approved the final version of the manuscript.

## Funding

The World Cancer Research Fund (WCRF), Grant 2006/11. the Commission of the European Communities, specific RTD programme 'Quality of Life and Management of Living Resources', QLK1-2001-00547 'Promoting and Sustaining Health through Increased Vegetable and Fruit Consumption among European Schoolchildren' (Pro Children).
